# Supplementary material for: Modeling glioblastoma heterogeneity as a dynamic network of cell states
Source: Mol Syst Biol. 2021 Sep 16;17(9):e10105. doi: 10.15252/msb.202010105 (PMC8444284; doi:10.15252/msb.202010105)
Supplement: Supplementary file 6 — Source Data for Figure 5 [file MSB-17-e10105-s004.zip › Figure5A_sourcedata/GSEA_3071/nosigdb_stateI.GseaPreranked.1622795145664/FELDMAN_2020_G1.html]

Details for gene set FELDMAN\_2020\_G1[GSEA]

|  || Dataset | state13071 |
| Phenotype | NoPhenotypeAvailable |
| Upregulated in class | na\_neg |
| GeneSet | FELDMAN\_2020\_G1 |
| Enrichment Score (ES) | -0.59177595 |
| Normalized Enrichment Score (NES) | -3.2580068 |
| Nominal p-value | 0.0 |
| FDR q-value | 0.0 |
| FWER p-Value | 0.0 |
Table: GSEA Results Summary

  

Fig 1: Enrichment plot: FELDMAN\_2020\_G1      
 Profile of the Running ES Score & Positions of GeneSet Members on the Rank Ordered List

  

| PROBE | GENE SYMBOL | GENE\_TITLE | RANK IN GENE LIST | RANK METRIC SCORE | RUNNING ES | CORE ENRICHMENT || 1 | GJB2 |  |  | 8 | 1.906 | 0.0170 | No |
| 2 | MIAT |  |  | 41 | 1.422 | 0.0219 | No |
| 3 | TPST2 |  |  | 56 | 1.325 | 0.0312 | No |
| 4 | CTGF |  |  | 152 | 0.913 | 0.0123 | No |
| 5 | IGFBP5 |  |  | 198 | 0.833 | 0.0074 | No |
| 6 | AHNAK2 |  |  | 220 | 0.786 | 0.0091 | No |
| 7 | MALAT1 |  |  | 1252 | 0.276 | -0.2937 | No |
| 8 | CYR61 |  |  | 1318 | 0.265 | -0.3102 | No |
| 9 | CCDC34 |  |  | 1376 | 0.255 | -0.3245 | No |
| 10 | BUB3 |  |  | 1687 | -0.278 | -0.4136 | No |
| 11 | CCNB2 |  |  | 1757 | -0.288 | -0.4311 | No |
| 12 | CALM2 |  |  | 1763 | -0.289 | -0.4297 | No |
| 13 | HNRNPA2B1 |  |  | 1819 | -0.296 | -0.4430 | No |
| 14 | TROAP |  |  | 1821 | -0.296 | -0.4403 | No |
| 15 | RAN |  |  | 1907 | -0.306 | -0.4624 | No |
| 16 | KPNA2 |  |  | 1944 | -0.312 | -0.4699 | No |
| 17 | DDX39A |  |  | 2043 | -0.324 | -0.4956 | No |
| 18 | SNRPB |  |  | 2064 | -0.328 | -0.4983 | No |
| 19 | HNRNPDL |  |  | 2142 | -0.342 | -0.5176 | No |
| 20 | HSP90AA1 |  |  | 2156 | -0.344 | -0.5180 | No |
| 21 | PLK1 |  |  | 2206 | -0.353 | -0.5289 | No |
| 22 | CENPA |  |  | 2220 | -0.357 | -0.5291 | No |
| 23 | NUCKS1 |  |  | 2278 | -0.368 | -0.5423 | No |
| 24 | TUBB4B |  |  | 2291 | -0.370 | -0.5421 | No |
| 25 | CKS1B |  |  | 2332 | -0.380 | -0.5501 | No |
| 26 | CKAP2 |  |  | 2357 | -0.385 | -0.5533 | No |
| 27 | PSIP1 |  |  | 2364 | -0.386 | -0.5512 | No |
| 28 | CENPW |  |  | 2402 | -0.393 | -0.5582 | No |
| 29 | H2AFV |  |  | 2431 | -0.398 | -0.5624 | No |
| 30 | CKS2 |  |  | 2466 | -0.404 | -0.5684 | No |
| 31 | AURKA |  |  | 2467 | -0.404 | -0.5643 | No |
| 32 | CDC20 |  |  | 2540 | -0.425 | -0.5813 | No |
| 33 | H2AFZ |  |  | 2572 | -0.433 | -0.5861 | No |
| 34 | TMEM106C |  |  | 2592 | -0.437 | -0.5873 | Yes |
| 35 | ANP32B |  |  | 2593 | -0.438 | -0.5829 | Yes |
| 36 | C12orf75 |  |  | 2607 | -0.441 | -0.5823 | Yes |
| 37 | SRSF7 |  |  | 2619 | -0.445 | -0.5810 | Yes |
| 38 | DUT |  |  | 2640 | -0.450 | -0.5824 | Yes |
| 39 | EXOSC8 |  |  | 2659 | -0.456 | -0.5831 | Yes |
| 40 | CCNA2 |  |  | 2667 | -0.462 | -0.5805 | Yes |
| 41 | KIF22 |  |  | 2683 | -0.468 | -0.5802 | Yes |
| 42 | CMC2 |  |  | 2694 | -0.470 | -0.5784 | Yes |
| 43 | SNRPD1 |  |  | 2701 | -0.471 | -0.5754 | Yes |
| 44 | ANLN |  |  | 2702 | -0.472 | -0.5706 | Yes |
| 45 | HAT1 |  |  | 2703 | -0.472 | -0.5658 | Yes |
| 46 | PRC1 |  |  | 2705 | -0.472 | -0.5613 | Yes |
| 47 | CENPN |  |  | 2708 | -0.474 | -0.5571 | Yes |
| 48 | KNSTRN |  |  | 2722 | -0.478 | -0.5561 | Yes |
| 49 | NDC80 |  |  | 2723 | -0.478 | -0.5513 | Yes |
| 50 | CYCS |  |  | 2727 | -0.479 | -0.5473 | Yes |
| 51 | RPA3 |  |  | 2730 | -0.480 | -0.5430 | Yes |
| 52 | CENPF |  |  | 2761 | -0.490 | -0.5470 | Yes |
| 53 | NUF2 |  |  | 2781 | -0.497 | -0.5475 | Yes |
| 54 | TUBA1B |  |  | 2785 | -0.498 | -0.5434 | Yes |
| 55 | DTYMK |  |  | 2798 | -0.501 | -0.5419 | Yes |
| 56 | HMGB1 |  |  | 2806 | -0.503 | -0.5388 | Yes |
| 57 | LSM5 |  |  | 2820 | -0.509 | -0.5375 | Yes |
| 58 | SIVA1 |  |  | 2822 | -0.510 | -0.5327 | Yes |
| 59 | ARL6IP1 |  |  | 2823 | -0.510 | -0.5275 | Yes |
| 60 | CDCA3 |  |  | 2829 | -0.513 | -0.5238 | Yes |
| 61 | HMGB3 |  |  | 2839 | -0.516 | -0.5212 | Yes |
| 62 | TOP2A |  |  | 2842 | -0.517 | -0.5166 | Yes |
| 63 | CEP55 |  |  | 2852 | -0.522 | -0.5139 | Yes |
| 64 | CKAP2L |  |  | 2857 | -0.523 | -0.5098 | Yes |
| 65 | NUDT1 |  |  | 2859 | -0.524 | -0.5048 | Yes |
| 66 | RAD21 |  |  | 2866 | -0.527 | -0.5012 | Yes |
| 67 | ANP32E |  |  | 2877 | -0.530 | -0.4988 | Yes |
| 68 | PTTG1 |  |  | 2895 | -0.540 | -0.4984 | Yes |
| 69 | PRCP |  |  | 2919 | -0.553 | -0.4996 | Yes |
| 70 | CENPH |  |  | 2945 | -0.569 | -0.5012 | Yes |
| 71 | MIS18A |  |  | 2947 | -0.570 | -0.4958 | Yes |
| 72 | KIF4A |  |  | 2953 | -0.574 | -0.4914 | Yes |
| 73 | KIF23 |  |  | 2961 | -0.579 | -0.4876 | Yes |
| 74 | MZT1 |  |  | 2964 | -0.580 | -0.4823 | Yes |
| 75 | GGH |  |  | 2975 | -0.585 | -0.4794 | Yes |
| 76 | VRK1 |  |  | 2983 | -0.592 | -0.4754 | Yes |
| 77 | SMC2 |  |  | 2984 | -0.592 | -0.4694 | Yes |
| 78 | TPX2 |  |  | 3006 | -0.606 | -0.4695 | Yes |
| 79 | DNMT1 |  |  | 3035 | -0.622 | -0.4715 | Yes |
| 80 | LSM3 |  |  | 3037 | -0.625 | -0.4655 | Yes |
| 81 | DLG1 |  |  | 3043 | -0.628 | -0.4606 | Yes |
| 82 | AURKB |  |  | 3056 | -0.639 | -0.4577 | Yes |
| 83 | PHF19 |  |  | 3060 | -0.643 | -0.4520 | Yes |
| 84 | MAP3K7CL |  |  | 3076 | -0.657 | -0.4498 | Yes |
| 85 | GTSE1 |  |  | 3083 | -0.663 | -0.4449 | Yes |
| 86 | TMPO |  |  | 3088 | -0.666 | -0.4393 | Yes |
| 87 | MAD2L1 |  |  | 3099 | -0.675 | -0.4354 | Yes |
| 88 | DBF4 |  |  | 3117 | -0.690 | -0.4335 | Yes |
| 89 | LMNB1 |  |  | 3121 | -0.692 | -0.4273 | Yes |
| 90 | SMC4 |  |  | 3126 | -0.699 | -0.4214 | Yes |
| 91 | CENPK |  |  | 3127 | -0.700 | -0.4144 | Yes |
| 92 | SCG2 |  |  | 3130 | -0.703 | -0.4078 | Yes |
| 93 | GMNN |  |  | 3140 | -0.719 | -0.4032 | Yes |
| 94 | CDK1 |  |  | 3149 | -0.727 | -0.3982 | Yes |
| 95 | MKI67 |  |  | 3158 | -0.738 | -0.3931 | Yes |
| 96 | ORC6 |  |  | 3159 | -0.738 | -0.3856 | Yes |
| 97 | TK1 |  |  | 3162 | -0.745 | -0.3786 | Yes |
| 98 | DNAJC9 |  |  | 3167 | -0.747 | -0.3722 | Yes |
| 99 | STMN1 |  |  | 3171 | -0.751 | -0.3655 | Yes |
| 100 | USP1 |  |  | 3183 | -0.762 | -0.3611 | Yes |
| 101 | CDCA2 |  |  | 3189 | -0.767 | -0.3548 | Yes |
| 102 | BIRC5 |  |  | 3208 | -0.793 | -0.3521 | Yes |
| 103 | FBXO5 |  |  | 3212 | -0.797 | -0.3449 | Yes |
| 104 | RRM1 |  |  | 3215 | -0.801 | -0.3373 | Yes |
| 105 | SPC25 |  |  | 3219 | -0.806 | -0.3301 | Yes |
| 106 | UBE2C |  |  | 3225 | -0.812 | -0.3233 | Yes |
| 107 | NUSAP1 |  |  | 3237 | -0.825 | -0.3182 | Yes |
| 108 | HMGN2 |  |  | 3242 | -0.833 | -0.3109 | Yes |
| 109 | CENPM |  |  | 3269 | -0.881 | -0.3097 | Yes |
| 110 | FEN1 |  |  | 3278 | -0.890 | -0.3031 | Yes |
| 111 | H2AFX |  |  | 3282 | -0.898 | -0.2948 | Yes |
| 112 | RANBP1 |  |  | 3290 | -0.911 | -0.2877 | Yes |
| 113 | CDKN3 |  |  | 3293 | -0.916 | -0.2790 | Yes |
| 114 | ZWINT |  |  | 3296 | -0.925 | -0.2702 | Yes |
| 115 | PCNA |  |  | 3319 | -0.963 | -0.2670 | Yes |
| 116 | UBE2T |  |  | 3330 | -0.979 | -0.2600 | Yes |
| 117 | DEK |  |  | 3344 | -1.025 | -0.2535 | Yes |
| 118 | PBK |  |  | 3354 | -1.046 | -0.2455 | Yes |
| 119 | ESCO2 |  |  | 3367 | -1.088 | -0.2380 | Yes |
| 120 | NCAPG |  |  | 3370 | -1.098 | -0.2275 | Yes |
| 121 | MCM7 |  |  | 3384 | -1.147 | -0.2197 | Yes |
| 122 | GINS2 |  |  | 3386 | -1.156 | -0.2083 | Yes |
| 123 | DHFR |  |  | 3387 | -1.159 | -0.1965 | Yes |
| 124 | HMGB2 |  |  | 3390 | -1.184 | -0.1851 | Yes |
| 125 | CLSPN |  |  | 3391 | -1.184 | -0.1731 | Yes |
| 126 | UBE2S |  |  | 3396 | -1.205 | -0.1621 | Yes |
| 127 | CENPU |  |  | 3398 | -1.230 | -0.1499 | Yes |
| 128 | HIST1H1E |  |  | 3401 | -1.237 | -0.1380 | Yes |
| 129 | ATAD2 |  |  | 3415 | -1.278 | -0.1289 | Yes |
| 130 | RRM2 |  |  | 3420 | -1.297 | -0.1169 | Yes |
| 131 | HIST1H1C |  |  | 3427 | -1.335 | -0.1051 | Yes |
| 132 | IGFBP3 |  |  | 3433 | -1.384 | -0.0926 | Yes |
| 133 | TYMS |  |  | 3436 | -1.391 | -0.0791 | Yes |
| 134 | HIST1H1B |  |  | 3467 | -1.770 | -0.0700 | Yes |
| 135 | PTN |  |  | 3476 | -1.843 | -0.0537 | Yes |
| 136 | SCRG1 |  |  | 3480 | -1.876 | -0.0356 | Yes |
| 137 | S100B |  |  | 3489 | -2.109 | -0.0166 | Yes |
| 138 | HIST1H4C |  |  | 3493 | -2.248 | 0.0053 | Yes |
Table: GSEA details [plain text format]

  

Fig 2: FELDMAN\_2020\_G1: Random ES distribution      
 Gene set null distribution of ES for **FELDMAN\_2020\_G1**
